# Korean Red Ginseng Extract Attenuates 3-Nitropropionic Acid-Induced Huntington's-Like Symptoms

**DOI:** 10.1155/2013/237207

**Published:** 2013-01-27

**Authors:** Minhee Jang, Min Jung Lee, Cheon Suk Kim, Ik-Hyun Cho

**Affiliations:** ^1^Department of Anatomy, College of Korean Medicine and Institute of Korean Medicine, Kyung Hee University, Seoul 130-701, Republic of Korea; ^2^Department of Cancer Preventive Material Development and Institute of Korean Medicine, Kyung Hee University, Seoul 130-701, Republic of Korea; ^3^Analysis Research Team, R&D Headquarters, Korea Ginseng Corporation, Daejeon 305-805, Republic of Korea

## Abstract

Korean red ginseng (KRG) possesses neuroprotective activity. However, the potential neuroprotective value of KRG for the striatal toxicity is largely unknown. We investigated whether KRG extract (KRGE) could have a neuroprotective effect in a 3-nitropropionic acid- (3-NP) induced (i.p.) Huntington's disease (HD) model. KRGE (50, 100, and 250 mg/kg/day, p.o.) was administrated 10 days before 3-NP injection (pre-administration), from the same time with 3-NP injection (co-administration), or from the peak point of neurological impairment by 3-NP injection (post-administration). Pre-administration of KRGE produced the greatest neuroprotective effect in this model. Pre-administration of KRGE significantly decreased 3-NP-induced neurological impairment, lethality, lesion area, and neuronal loss in the 3-NP-injected striatum. KRGE attenuated microglial activation and phosphorylation of mitogen-activated protein kinases (MAPKs) and nuclear factor-kappa B (NF-**κ**B) signal pathway. KRGE also reduced the level of mRNA expression of tumor necrosis factor-alpha, interleukin- (IL-) 1**β**, IL-6, inducible nitric oxide synthase, and OX-42. Interestingly, the intrathecal administration of SB203580 (a p38 inhibitor) or PD98059 (an inhibitor of MAPK Kinase, MEK) increased the survival rate in the 3-NP-induced HD model. Pre-administration of KRGE may effectively inhibit 3-NP-induced striatal toxicity via the inhibition of the phosphorylation of MAPKs and NF-**κ**B pathways, indicating its therapeutic potential for suppressing Huntington's-like symptoms.

## 1. Introduction

Huntington's disease (HD) is an inherited neurodegenerative monogenic disorder that causes progressive degeneration of cells in the brain. HD is characterized clinically by involuntary abnormal movements, psychiatric disturbance, and cognitive deficits and pathologically by degeneration of the gamma-aminobutyric acid ergic(GABA) medium-size spiny neurons [1]. HD originated as a mutation of the gene encoding the huntingtin- (*Htt-*) protein. The underlying genetic mutation has been identified as a CAG-repeat expansion in the IT15 gene of chromosome 4 [2]. The formation of mutant *Htt*-protein leads to oxidative stress, mitochondrial dysfunction, apoptosis, energy metabolism defects, excitotoxicity, and RNA dysregulation in neurons [3, 4]. However, the exact pathogenesis of striatal neuronal death in HD is as yet unclear [5]. Furthermore, there are no current therapies proven to prevent, ameliorate, or abrogate the disease process in HD.

Recent evidence suggests that noncell autonomous neurodegeneration may play a significant role in the progression of neurodegenerative disorders, including HD. Glial cells (microglia and astrocytes) have a dual role as an integral part of HD pathogenesis [2, 3, 6, 7]. Before the clinical onset of HD patients and animal models of the disease, innate immune-response cells such as microglia, macrophages, and monocytes are activated at an early stage [4, 8–10]. During the progression of HD, activated microglia release neurotrophic mediators that include nerve growth factor (NGF), glial cell line-derived neurotrophic factor (GDNF), ciliary neurotrophic factor (CNTF), and transforming growth factor-beta (TGF-*β*), which have beneficial effects (i.e., trophic activity and phagocytosis) in neurodegenerative lesions. Also, activated microglia increase neuronal damage by secreting neurotoxic mediators that include tumor necrosis factor-alpha (TNF-*α*), interleukin- (IL-) 1*β*, nitric oxide (NO), prostaglandin E2 (PGE-2), hydrogen peroxide (H_2_O_2_), and superoxide [6, 7]. Therefore, inflammatory microglial activation is regarded as an attractive therapeutic target for the treatment of various neurological disorders, including HD, that accompany neuronal cell death [6].

Mitogen-activated protein kinases (MAPKs) and nuclear factor kappa-light-chain-enhancer of activated B cells (NF-*κ*B) signaling pathways are upstream cascades in inflammatory reactions [11, 12]. The MAPK superfamily is composed of three major signaling pathways: c-Jun NH_2_-terminal kinase (JNK), extracellular signal-regulated kinase (ERK) 1/2, and p38 kinase [11, 13, 14]. MAPKs are differentially activated in different experimental models of neurological disorders, including HD; play distinct roles in regulating synaptic plasticity, gene expression, neuronal apoptosis, and neuronal survival; and have emerged as targets for neurodegenerative diseases [11, 13, 14]. Basal levels of both activated JNK and p38 were elevated in the striatum from presymptomatic yeast artificial chromosome (YAC) 128 transgenic mice [15]. JNK is activated in striatal neuronal cells and in hippocampal cells by 3-NP [16–19] or mutation *Htt* intoxication [20]. ERK is activated in a more complex manner and may protect or damage neuronal cells [20–22]. It has recently been reported that ERK activation protects against mutant *Htt*-associated toxicity [20]. p38 is activated in the core region with striatal neuronal death within 1 hour of quinolinic acid injection [23] and in striatal neurons of R6/2 mice at a late stage in HD [24]. p38 is involved in enhanced NMDA receptor-dependent excitotoxicity in a YAC128 transgenic mouse model of HD [15]. In the central nervous system (CNS), the NF-*κ*B pathway is activated by cytokines, neurotrophic factors, neurotransmitters, injury, seizure, and proteins implicated in neurodegenerative disorders [12]. NF-*κ*B activation is upregulated by 3-NP or kainate intoxication in the striatum, which mimics pathology caused by mutant *Htt* [25]. Based on these data, it is reasonable to suggest that pharmacological modulation of the MAPKs and NF-*κ*B pathway may provide a new therapeutic target in HD.

*Panax (P.) ginseng *C. A. Meyer, a perennial herb of the family *Araliaceae,* has been traditionally used (over 2,000 years) as a medicinal preparation in Republic of Korea, China, and Japan. The basis of the medicinal prowess remains unknown. Its roots and extracts have been used to increase physical strength, prevent aging, and increase vigor [26]. The major active ingredients of *P*. *ginseng* are the triterpene glycosides also known as ginsenosides, which contain an aglycone with a dammarane skeleton [27]. It has recently been demonstrated that *P*. *ginseng* and ginsenosides produce immune, endocrine, cardiovascular, and cancer-related benefits [26, 28–31]. Also, *P*. *ginseng* and ginsenosides have protective actions in CNS disorders including Parkinson's disease (PD) [32–34], Alzheimer's disease (AD) [32, 35, 36], HD [5, 32, 37, 38], and stroke [32, 39, 40].

Total saponins (GTS) and ginsenosides have protective effects against neurotoxin insults [15, 37, 38, 41–44]. GTS have protective effects against 3-nitropropionic acid- (3-NP-) induced striatal degeneration via inhibition of intracellular Ca^2+^ elevations [37]. Pretreatment with Rb1, Rc, and Rg5 effectively protects YAC128 medium spiny striatal neurons (MSN) from glutamate-induced apoptosis by inhibiting glutamate-induced Ca^2+^ responses [38]. Also, GTS and Rh1 have anti-inflammatory activity in lipopolysaccharide- (LPS-) stimulated microglia [41, 42]. Interestingly, GTS and ginsenosides (Rh1, Rb1) have anti-inflammatory effects by the regulating the MAPKs and NF-*κ*B pathways in the LPS-stimulated microglia [41], H_2_O_2_-induced neuronal toxicity [15], and cerebral ischemic brain [43, 44].

Based on these reports, we studied whether Korean red ginseng (KRG; *Ginseng Radix Rubra*), the steamed root of *P*. *Ginseng*, has a neuroprotective effect in 3-NP-stimulated striatal toxicity by controlling the microglial activation, and the MAPKs, and NF-*κ*B pathways. In the present study, we confirmed that preadministration of KRGE improved the clinical behavior, striatal neuronal death by inhibiting microglial activation, activation of the pathways of the JNK, ERK, and p38 MAPKs and NF-*κ*B and the expression of proinflammatory cytokine (TNF-*α*, IL-6, and IL-1*β*) and iNOS.

## 2. Materials and Methods

### 2.1. Animals and Ethical Approval

The male ICR mice (weight, 20–25 g) were kept at a constant temperature of 23 ± 2°C with a 12-hour light-dark cycle (light on 08:00 to 20:00), and fed food and water *ad libitum*. The animals were allowed to habituate to the housing facilities for 1 week before the experiments. All experimental procedures were reviewd and approved by the Institutional Animal Care and Use Committee (IACUC) at Kyung Hee University.

### 2.2. KRGE and Administration

KRG extract (KRGE; Hong Sam Jung) was manufactured from roots of 6-year-old fresh ginseng, *P*. *ginseng* C. A. Meyer, which was harvested in Republic of Korea by Korea Ginseng Corporation (Daejeon, Republic of Korea). KRG was made by steaming the fresh ginseng roots at 90–100°C for 3 hours and then drying at 50–80°C. KRGE was prepared from KRG water extract, which was extracted in three 8-hour cycles of circulating hot water (85–90°C). The contents of moisture, crude protein, crude saponin, carbohydrate, and crude ash were analyzed at Korea Ginseng Corporation according to the method outlined in the Korea Food Code [45] (Table 1). Also, KRGE was analyzed by high-performance liquid chromatography. KRGE contained major ginsenoside-Rb1 (5.89 mg/g), -Rb2 (2.30 mg/g), -Rc (2.78 mg/g), -Rd (0.92 mg/g), -Re (1.16 mg/g), -Rf (1.00 mg/g), -Rg1 (0.96 mg/g), -Rg2s (1.42 mg/g), -Rg3r (1.16 mg/g), -Rg3s (2.41 mg/g), and -Rh1 (0.96 mg/g) and other minor ginsenosides.

### 2.3. Administration of 3-NP and Behavioral Assessment

**Figure 1:**
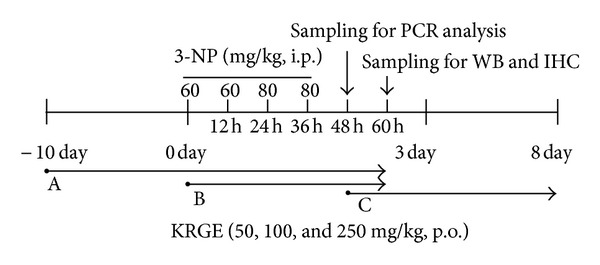
Schematic drawing of the experimental protocols used for intoxication with 3-NP. Behavioral testing was conducted twice a day until the end of the experiment. A: preadministration (from 10 days before first 3-NP injection); B: coadministration (from the same day with first 3-NP injection); C: postadministration (from the peak day of neurological impairment by 3-NP injection); WB: western blot; IHC: immunohistochemistry; 3-NP: 3-nitropropionic acid; KRGE: Korea red ginseng extract.

Mice were divided into three experimental groups (Figure 1) and each experimental group was subdivided into the following six groups (Supplementary Data 1 available online at http://dx.doi.org/10.1155/2013/237207): normal (saline, intraperitoneally (i.p.) + saline, per os (p.o.)), 3-NP + saline (60-60-80-80 mg/kg of 3-NP, i.p. + saline, p.o.), 3-NP + KRGE 50, 100, and 250 (60-60-80-80 mg/kg of 3-NP, i.p. + 50, 100, and 250 mg/kg of KRGE, p.o.), and saline + KRGE 250 group (saline, i.p. + 250 mg/kg of KRGE, p.o.). Each experiment was repeated more than three times using the same protocol. 3-NP was dissolved in saline to a concentration of 50 mg/mL (pH 7.4) and passed through a 0.2 *μ*m filter to remove any bacteria and kept at −80°C until use. 3-NP solution was administered i.p. twice daily for 2 days at 12-hour intervals (9:00 AM and 9:00 PM) at a dose of 60 mg/kg on the first day and 80 mg/kg on the second day (i.e., a 60-60-80-80 dose regimen) [46] (Figure 1).

**Figure 2:**
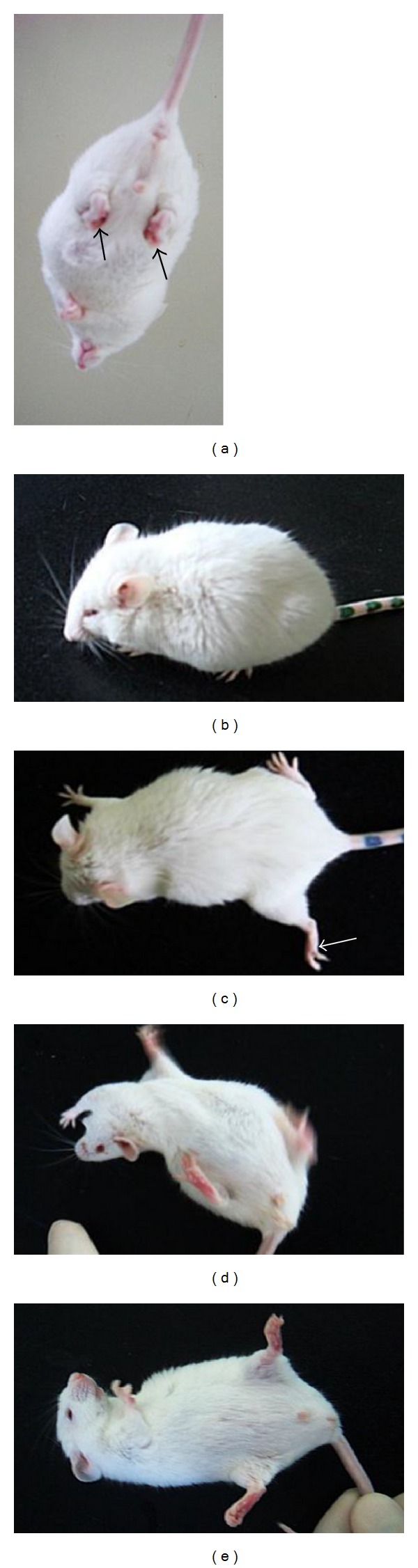
3-NP-induced motor and postural abnormalities at day 1 after intoxication. (a) Hindlimb clasping (arrow). (b) Truncal dystonia (kyphotic posture). (c) Hindlimb dystonia (arrow). (d-e) Impaired postural adjustments following postural challenge; the mouse is tipped up on its side (d) and cannot get back on its feet on its own (e).

We adopted a previously described behavioral score [47] to provide optimal assessment of neurological behavioral deficits after 3-NP administration. Briefly, the three-level scale assessed the severity of the following five items (maximal score = 10), which constitute the main motor symptoms observed: hindlimb clasping, reduced global activity in a freely moving environment, hindlimb dystonia, truncal dystonia (kyphosis), and balance adjustments to a postural challenge (Figure 2). Mice were rated before each injection (every 12 hours) during the intoxication procedure, then each day for 1 or 5 days after intoxication. The behavioral test was also performed by an experimenter who was unaware of the experimental condition under constant conditions of temperature (23 ± 3°C) and humidity (55 ± 5%) in a quiet room.

### 2.4. Histological Assessment of Striatal Damage

To evaluate the histopathological change, 24 hours after the last 3-NP injection, the mice were anesthetized by sodium pentobarbital (30 mg/kg, body weight, i.p.) and then perfused intracardially with saline, followed by cold 4% paraformaldehyde (PFA) in 0.1 M phosphate buffer (PB, pH 7.4). The brains were immediately removed, postfixed overnight in the same fixative at 4°C, rinsed twice with PBS, and cryoprotected in 10, 20, and 30% sucrose in 0.1 M phosphate-buffered saline (PBS, pH 7.4) serially for 48 hours at 4°C. Sequential coronal sections (30 *μ*m thickness) were acquired on a model CM3050S freezing microtome (Leica, Germany), starting from the anterior aspect of the corpus callosum throughout the entire striatum (bregma 1.40 ~ −1.30 mm), according to the mouse brain atlas [48]. Sections were collected in sequence as free-floating sections on PBS and stored at −20°C until use for histochemical studies. For histological assessment, every tenth section (at intervals of 300 *μ*m) was processed for cresyl violet staining and dehydrated in an ascending ethanol series, immersed in xylene, and coverslipped with Permount (Fisher Scientific, USA). The stained sections from the level of the midstriatum were captured using a DP70 image analysis system (Olympus, Japan) and the level of 3-NP-induced striatal damage was analyzed using NIH Image J program (http://rsbweb.nih.gov/ij/).

### 2.5. Immunohistochemical Evaluation

Immunohistochemical analysis was performed as previously described [49]. Briefly, brain sections were incubated for 30 minutes with 3% H_2_O_2_ in 0.1 M PBS (pH 7.4) to remove endogenous peroxidase activity and washed in PBS. Sections were then blocked with a solution containing 5% normal goat/or horse serum, 2% bovine serum albumin, 2% fetal bovine serum, and 0.1% triton X-100 for 2 hours at room temperature (RT). The sections were incubated overnight at 4°C with either mouse anti-NeuN (1 : 1,000; Chemicon, USA), rabbit anti-Iba-1 (1 : 2,000; WAKO, Japan), rabbit anti-glial fibrillary acidic protein (GFAP) (1 : 5,000; DACO, USA), or rabbit anti-caspase-3 antibody (1 : 1,000; Cell Signaling Technology, USA). And sections were incubated overnight at 4°C and washed in PBS. Sections were then incubated with biotinylated rabbit/mouse IgG antibody (1 : 200; Vector Laboratories, USA) for 1 hour at RT. After rinsing, the sections were incubated with avidin-biotinylated horseradish peroxidase- (HRP-) complex (1 : 200; Vector Laboratories, USA) for 1 hour at RT and visualized with 3,3′-diamino-benzidine (DAB). Sections were rinsed, and dehydrated and cover-slipped with Permount. To perform quantitative analysis of NeuN-immunoreactivity, three or four sections per animal were selected, one or two fields (×200 magnification) per region of interesting of each section were captured, and the captured images were semiautomatically analyzed using the NIH Image J program. The entire quantifying procedure was blindly performed.

### 2.6. Western Blot Analysis

To investigate the level of p-JNK, p-ERK, p-p38, NF-*κ*B, and caspase-3 expression, 24 hours after the last injection of 3-NP, the mice (*n* = 4/group) were anesthetized, and the striatums were removed with lysis buffer (50 mM Tris-Cl, pH 7.5, 150 mM NaCl, 1% Triton X-100, 10% glycerol, and protease inhibitor mixture). A total of 30 *μ*g of tissue lysate from each sample was resolved by 10% SDS-PAGE. The proteins were then transferred to polyvinylidene fluoride membranes and blocked with 5% nonfat dry milk in Tween-20-containing Tris-buffered saline (TBST, 20 mM Tris, pH 7.4, 0.1% Tween 20, and 150 mM NaCl). The membranes were probed overnight with rabbit anti-p-JNK, p-ERK, p-p38 (1 : 1,000; Cell Signaling Technology, USA), rabbit anti-NF-*κ*B (1 : 1,000; Santa Cruz Biotechnology, USA), or rabbit anti-caspase-3 (1 : 1,000) antibody at 4°C, which was followed by incubation with HRP-conjugated secondary antibody at RT for 1 hour prior to enhanced chemiluminescence (Amersham Pharmacia Biotech, USA) treatment and exposure to X-ray film. For normalization of antibody signal, the membranes were stripped and reprobed with antibodies for JNK, ERK 1/2, p38 (1 : 2,000; Cell Signaling Technology, USA) or actin (1 : 2,000; Santa Cruz Biotechnology, USA). After western blot was performed several times, the density of each band was converted to numerical values using the Photoshop CS2 program (Adobe, USA), subtracting background values from an area of film immediately adjacent to the stained band. Data are expressed as the ratio of p-JNK, p-ERK, p-p38, and NF-*κ*B against total JNK, ERK 1/2, p38, and actin for each sample.

### 2.7. Real-Time Polymerase Chain Reaction (PCR) and Reverse-Transcription- (RT-) PCR Analyses

To investigate the mRNA level of proinflammatory cytokines and chemokines, 12 hours after the last injection of 3-NP, the mice used for real-time PCR analysis (*n* = 4/group) were anesthetized, and each striatum was removed and deep-frozen. Real-time PCR was performed using SYBR Green PCR Master Mix (Applied Biosystems, USA) as previously described [49]. Reactions were performed in duplicate in a total volume of 10 *μ*L, each containing 10 pM primer, 4 *μ*L cDNA and 5 *μ*L SYBR Green PCR Master Mix. The mRNA levels of each target gene were normalized to that of glyceraldehyde 3-phosphate dehydrogenase (GAPDH) mRNA. Fold induction was calculated using the 2^−ΔΔCT^ method as previously described [50]. All real-time PCR experiments were performed at least three times, and the mean ± SEM values are presented unless otherwise noted. The primer sequence information is provided in Table 2. For RT-PCR analysis, total RNA was isolated from striatum using TriZol reagent according to the manufacturer's instructions (Invitrogen, USA). cDNA was synthesized by incubating 1 *μ*g of total RNA for 1 hour at 37°C in a reaction mixture, containing 0.5 *μ*g of Oligo dT, 0.5 mM dNTP mix, 5× first-strand buffer, RNase out, 5 mM dithiothreitol (DTT), and M-MLV reverse transcriptase. PCR analysis was performed according to the manufacturer's instructions (RT-PCR kit; Roche, Germany) with the primers summarized in Table 2. Saturation curves for PCR were obtained from various experimental conditions (RNA concentrations, annealing temperatures, and PCR cycle numbers). We determined the optimal amplification conditions (annealing temperature and PCR cycle number) of primers for the PCR. Products were electrophoresed on a 1% agarose gel and visualized by staining with ethidium bromide [49].

### 2.8. Intrathecal (i.t.) Administration of SB203580, PD98059, and Ginsenosides

The i.t. injections were performed under light isoflurane anesthesia (1-2%). The dorsocervical fur of each mice (vehicle + saline, *n*  =  6; vehicle + 3-NP, *n*  =  8; 1.0 *μ*g of SB203580 + 3-NP, *n*  =  8; 5.0 *μ*g of SB203580 + 3-NP, *n*  =  6; 0.1 *μ*g of PD98059 + 3-NP, *n*  =  7) was shaved, the spinal column was arched, and a 30-gauge needle was directly inserted into the subarachnoid space, between the L5 and L6 vertebrae [51, 52]. Correct i.t. positioning of the needle tip was confirmed by manifestation of a characteristic tail flick response. The 1 and 5 *μ*g of the p38 inhibitor SB203580 (Calbiochem, USA), the 0.1 *μ*g of the ERK upstream kinase (MAPK Kinase, MEK) inhibitor PD98059 (Calbiochem, USA), or vehicle (saline alone or 10% dimethylsulfoxide) were slowly injected into the mice with a 50 *μ*L Hamilton microsyringe in a total volume of 10 *μ*L. The entire injection procedure, from the induction of anesthesia until recovery of consciousness, took 4-5 minutes. Preliminary injections were performed with a similar volume of 10% India ink solution and the reliability and accuracy of this method was confirmed by subsequent dissection of the brain stem. The success rate for the prior injections with this technique was over 95.0%. The same investigator performed all injections. The i.p. injection of 3-NP was performed 20 minutes after i.t. injection of SB203580 and PD98059 as described above. Additionally, the i.t. introduction of ginsenoside-Rg1 and -Rb1 was performed as described above for the introduction of SB203580 and PD98059.

### 2.9. *In Situ* Detection of Fragmented DNA (Terminal Deoxynucleotidyl Transferase-Mediated UTP Nick End Labeling, TUNEL)

The fragmentation of DNA was examined using an ApopTag peroxidase *in situ* Apoptosis Detection Kit (S7100) (Millipore, USA) according to the manufacturer's instructions. Briefly, brain sections were placed to enzymatic digestion with a 20 *μ*g/mL of proteinase K for 5 minutes, treated with 5% H_2_O_2_ for 20 minutes to exhaust endogenous peroxidase activity, and washed with PBS (0.1 M, pH 7.4). They were then immersed in an ApopTag Equilibration Buffer to label the 3′-OH ends of fragmented DNA for 10 minutes and incubated with terminal deoxynucleotidyl transferase enzyme at 37°C for 1 hour. After washing with PBS, sections were incubated with anti-digoxygenin-conjugated peroxidase and the peroxidase substrate (DAB) to detect signs of apoptotic cell death.

### 2.10. Statistical Analysis

The statistical significance of differences between the values was determined using an ANOVA with a Fisher's post hoc test. All data are presented as the mean ± S.E.M., and a statistical difference was accepted at the 5% level unless indicated otherwise.

## 3. Results

### 3.1. Effects of KRGE on 3-NP-Induced Neurologic Impairments

#### 3.1.1. Preadministration of KRGE for 10 Days

**Figure 3:**
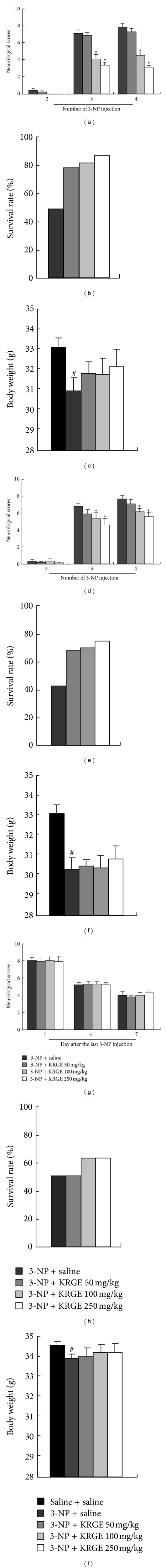
Changes of behavioral score, survival rate, and body weight during the 3-NP intoxication period and during recovery. The pre- (a–c) and coadministration (d–f) of KRGE reduced neurological impairment (a, d) and increased the survival rate (b, e) during 3-NP-intoxication period (a–f), but the postadministration of KRGE did not show positive effect during recovery (g–i). Statistical significance: *P < 0.05 versus 3-NP + saline-administrated mice; ^#^ *P* < 0.05 versus normal (saline + saline-administrated) mice.

First, we examined whether the preadministration of KRGE for 10 days could attenuate 3-NP-induced neurological impairments (Figure 3(a)). After the first two injections of 3-NP (60-60 mg/kg), 3-NP + saline-administrated, or 3-NP + KRGE- (50, 100, and 250 mg/kg) administrated mice did not display any visible neurological deficit symptoms. After the third injection of 3-NP (60-60-80 mg/kg), 3-NP + saline-administrated mice displayed clear neurological deficit symptoms including hindlimb clasping, reduced global activity in a freely moving environment, hindlimb dystonia, truncal dystonia (kyphosis), and balance adjustments to a postural challenge (behavioral score, 7.07 ± 0.37). After the fourth injection of 3-NP (60-60-80-80 mg/kg), most of the 3-NP + saline-administrated mice displayed more severe neurological deficit symptoms (behavioral score, 7.80 ± 0.39). In contrast, 3-NP + KRGE (100 and 250 mg/kg)-administratedmice displayed relatively slight neurological deficits compared to 3-NP + saline-administrated mice (Figure 3(a), Supplementary Data 1). Although the survival rate in 3-NP + saline-administrated mice was 48.4% at the end of the experiment, it was increased in dose-dependent manner by KRGE-administration (50 mg/kg, 77.8%; 100 mg/kg, 81.3%; 250 mg/kg, 86.7%) (Figure 3(b), Supplementary Data 1). Mean body weight was decreased in 3-NP + saline-administrated mice and was slightly increased in 3-NP + KRGE-administrated mice, although not significantly (Figure 3(c)).

#### 3.1.2. Coadministration of KRGE with 3-NP

Next, KRGE was administrated from the same day with first injection of 3-NP and we examined whether KRGE attenuated 3-NP-induced neurological impairments (Figure 3(d), Supplementary Data 1). The pattern of neurological deficit observed in the 3-NP + KRGE-administrated mice was essentially similar to those of preadministrated mice for 10 days (Figures 3(a) and 3(d), Supplementary Data 1). However, the extent of neurological deficit was higher than those of KRGE preadministrated mice (Figure 2(d), Supplementary Data 1). The survival rate in 3-NP + saline-administrated mice was 41.9% at the end of the experiment, and was increased in a dose-dependent manner by KRGE coadministration (50 mg/kg, 66.7%; 100 mg/kg, 68.8%; 250 mg/kg, 73.3%) (Figure 3(e)). However, coadministration of KRGE did not change mean body weight (Figure 3(f)).

#### 3.1.3. Postadministration of KRGE with 3-NP

Next, to investigate whether KRGE could recover 3-NP-induced neurological impairment, we administrated KRGE for 5 days from the peak day of neurological deficits induced by 3-NP-intoxication. Postadministration of KRGE did not remediate the motor function defects and mean body weight loss, but post-administration (100 and 250 mg/kg) of KRGE increased the survival rate at the end of the experiment (Figures 3(g)–3(i)). These data suggested that the pre- and coadministration of KRGE can reduce 3-NP-induced neurological impairment and survival rate, and that the preadministration of KRGE is more neuroeffective compared with that of co- and postadministrated mice.

### 3.2. Effects of KRGE on 3-NP-Induced Striatal Damage

**Figure 4:**
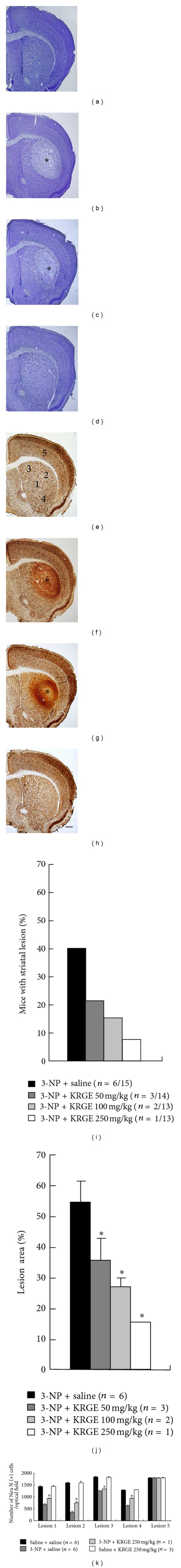
Preadministration of KRGE reduces striatal lesion volume and neuronal cell loss. (a–g) Representative photographs show the distribution of neuronal cell by cresyl violet staining (a–d) and immunohistochemistry for NeuN antibody (e–h). Asterisks indicate striatal lesions. Scale bar = 50 *μ*m. (i) Proportion of mice with striatal lesion was decreased by preadministration of KRGE (50, 100, and 250 mg/kg/day) in dose-dependent pattern. (j) Lesion area was significantly lower in the 3-NP + KRGE-administrated mice (50, 100, and 250 mg/kg/day) than in the 3NP + saline-administrated mice. (k) The number of NeuN(+) cells in the striatum of 3-NP + KRGE-administrated mice was increased than that in the 3-NP + saline-administrated mice. Statistical significance: *P < 0.05 versus 3-NP + saline-administrated mice.

In the above experiments, because preadministration of KRGE for 10 days was most effective in alleviating the development of 3-NP-induced neurological impairment, we investigated the connection between the extent of neurological deficit and the level of striatal damage. The level of 3-NP-induced striatal damage was examined 24 hours after the last 3-NP injection using cresyl violet staining.  Figure 4(a) shows representative striatal images from normal (saline + saline), 3-NP + saline-administrated, 3-NP + KRGE-administrated, and saline + KRGE-administrated mice. The staining demonstrated bilateral symmetrical lesions in the striatum of 3-NP + saline- and 3-NP + KRGE-administrated mice (Figures 4(a)–4(d)). Forty percent (*n* = 6) of surviving 3-NP-administrated mice (*n* = 15) had bilateral striatal lesions, whereas the ratio of mice with striatal lesion of surviving 3-NP + KRGE-administrated mice was decreased in a dose-dependent manner of KRGE (21.4% (*n* = 3/14) in 50 mg/kg, 15.3% (*n* = 2/13) in 100 mg/kg, and 7.7% (*n* = 1/13) in 250 mg/kg) (Figure 4(i), Supplementary Data 1). To quantify the degenerative striatum, we analyzed the mean lesion area in cresyl violet stained sections from the level of the midstriatum in which the degenerated striatum was present as a palely-stained area. The proportion of mean lesion area in the 3-NP + saline-administrated mice was 54.2 ± 6.8% for entire striatum, while that in the 3-NP + KRGE-administrated mice was 35.5 ± 7.1%, 26.9 ± 2.6%, and 13.5 ± 0.0% in 50, 100, and 250 mg/kg of KRGE, respectively, (Figure 4(j)).

Because preadministration of 250 mg/kg KRGE was the most effective for 3-NP-induced neurological impairment and striatal damage, we examined the immunohistochemical change in the neuron marker neuronal nuclear protein (NeuN) (Figures 4(e)–4(h) and 4(k)) in striatal sections from 250 mg/kg KRGE-administrated mice. The patterns of NeuN-immunoreactive (IR) neuronal degenerative areas observed in all groups of mice were consistent with the pale areas evident following by cresyl violet staining. 3-NP + saline-administrated striatum exhibited an extensive loss of the NeuN-IR area relative to lesion-free normal mice. However, preadministration of KRGE (250 mg/kg) significantly increased the NeuN-IR area in the striatum (Figures 4(e)–4(h)). The number of NeuN-IR neurons in the striatum was measured. At concentrations of 250 mg/kg, KRGE showed significant reductions in the loss of NeuN-IR neurons in several striatal lesions (1: whole striatum; 2: dorsolateral striatum; 3: dorsomedial striatum; 4: ventrolateral striatum; 34.5%, 114.9%, 10.2%, and 64.7%, resp.) relative to 3-NP + saline-administrated mice. However, the number of NeuN-IR neurons in the cortex was not affected by 3-NP or KRGE (Figure 4(k)). These histological data suggest that preadministration of KRGE exacerbates 3-NP-induced striatal damage.

### 3.3. Effects of KRGE on 3-NP-Induced Microglial Activation

**Figure 5:**
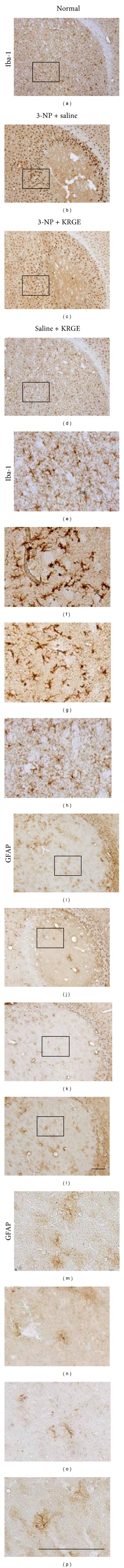
Suppression of the 3-NP-induced microglial activation, but not astrocyte, by KRGE preadministration. Striatal sections were stained with anti-Iba-1 (a–h) or anti-GFAP (i–p) antibodies 24 hours after 3-NP administration without and with KRGE preadministration (250 mg/kg/day, p.o.).(a–h) 3-NP-induced microglial activation was inhibited by KRGE preadministration. Panels (e–h) and (m–p) display high magnification micrographs of panels (a–d) and (i–l) marked with squares, respectively. Scale bar = 50 *μ*m.

**Figure 6:**
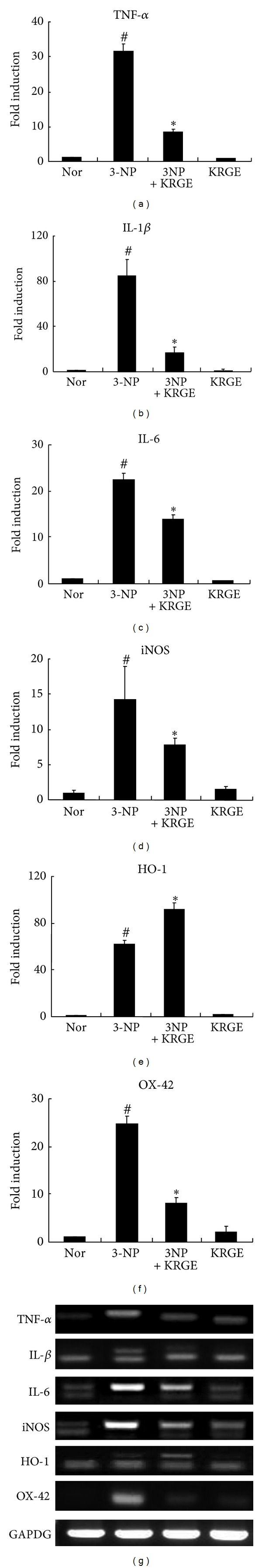
Suppression of the mRNA expression of 3-NP-induced inflammatory mediators by KRGE preadministration. Preadministration of KRGE (250 mg/kg/day, p.o.) inhibited the mRNA expression of proinflammatory cytokines (TNF-*α*, IL-1*β*, and IL-6), iNOS, and OX-42, while increasing HO-1 expression. Graphic data were performed by real-time PCR analysis (a–f). The bands show RT-PCR data (g). Statistical significance: ^#^ *P* < 0.05 versus normal (saline + saline-administrated mice); *P < 0.05 versus 3-NP + saline-administrated mice.

Microglia are activated in neurodegenerative diseases including HD, and activated microglia produce inflammatory mediators [6, 7]. KRGE attenuates inflammation through the inhibition of microglial activation in various neurological disease models [41, 42]. In the above experiments, preadministration of 250 mg/kg KRGE for 10 days produced the best protection against neurological impairment by 3-NP. Therefore, we examined whether preadministration of 250 mg/kg KRGE was capable of inhibiting microglial activation in a 3-NP-induced striatal damage model using immunohistochemistry. In the normal brain, Iba-1 (a marker for microglia/macrophage lineage cells) IR microglia generally displayed small cell bodies with thin processes (Figure 5(a)), which is the typical morphology of resting cells [49]. However, 3-NP-administration caused a marked change in cell shape to an enlarged cell body with short and thick processes (Figure 5(b)), a morphology consistent with an activated state [49]. Interestingly, preadministration of KRGE (250 mg/kg) inhibited Iba-1-IR microglial activation compared to that of 3-NP + saline-administrated mice (Figure 5(c)). In addition to morphological change of individual microglia, 3-NP significantly increased the expression of OX-42 mRNA compared to normal brain. However, preadministration of KRGE reduced its expression (Figures 6(f) and 6(g)). KRGE (250 mg/kg) itself did not induce microglial activation (Figures 5(d) and 5(h)). To examine if KRGE-administrated neuroprotective effect was correlated with blockade of microglial activation, Iba-1-immunoreactivity was confirmed in the brains of mice administrated 50 mg/kg KRGE, a dose that dose did not improve 3-NP-induced neurological impairment. Preadministration of 50 mg/kg KRGE had no effect on the inhibition of microglial reactivity (data not shown). GFAP-IR astrocytes were not activated in the striatum after 3-NP or KRGE treatment compared with normal mice (Figures 5(i)–5(p)). These results show that preadministration of KRGE suppresses 3-NP-induced microglia activation in the striatum and that microglia activation does not influence astrocyte activation.

### 3.4. Effects of KRGE on 3-NP-Induced Proinflammatory Cytokines

Because activated microglia produce inflammatory mediators implicated as putative neurotoxic and neurotrophic factors [6, 7], and because KRGE inhibits the expression of LPS-induced proinflammatory cytokines and enzyme inducible nitric oxide synthase (iNOS) in the brain, we investigated the effect of KRGE on expression of proinflammatory cytokines (TNF-*α*, IL-1*β*, and IL-6), iNOS, and HO-1 12 hours after the last 3-NP injection. Real-time PCR revealed little or no expression of TNF-*α*, IL-1*β*, IL-6, iNOS, and HO-1 mRNA in control stratum. However, 12 hours after last 3-NP injection, all inflammatory mediators were highly expressed (Figure 6). In contrast, animals preadministrated KRGE (250 mg/kg) for 10 days showed marked reductions in expression of TNF-*α*, IL-1*β*, IL-6, and iNOS mRNA compared with 3-NP + saline-administrated mice. KRGE itself had no effect on the increased expression of these factors (Figure 6). Relative to normal, i.p. 3-NP-administration caused significant induction of all factors: TNF-*α* (by 31-fold), IL-1*β* (by 84-fold), IL-6 (by 22-fold), and iNOS (by 14-fold). Preadministration of KRGE markedly reduced this 3-NP-induced expression of TNF-*α* (by 73%), IL-1*β* (by 80%), IL-6 (by 38%), and iNOS mRNA (by 45%) compared with 3-NP + saline-administrated mice. Interestingly, the expression of HO-1 mRNA was upregulated (by 32%) by preadministration of KRGE compared with 3-NP + saline-administrated mice (by 61-fold) (Figure 6).

### 3.5. Effects of KRGE on 3-NP-Induced MAPKs and NF-*κ*B Signaling Pathway

**Figure 7:**
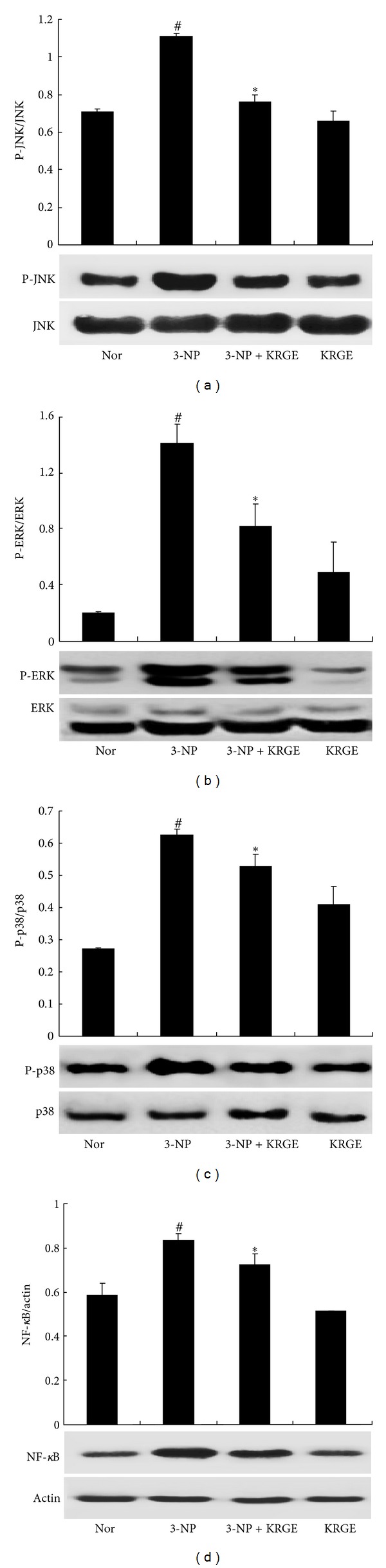
Preadministration of KRGE suppresses the activation of MAPKs and NF-*κ*B signal pathway. Preadministration of KRGE (250 mg/kg/day, p.o.) inhibited the activation of JNK (a), ERK (b), and p38 (c) MAPKs, and NF-*κ*B (d). Statistical significance: ^#^ *P* < 0.05 versus normal (saline + saline-administrated mice); *P < 0.05 versus 3-NP + saline-administrated mice.

MAPKs and NF-*κ*B are upstream signaling molecules in inflammatory reactions. To further investigate the anti-inflammatory mechanism of KRGE, the effects of KRGE on MAPKs and NF-*κ*B signaling pathways were examined using striatum 24 hours after last 3-NP + saline-administration. Western blot analysis revealed that 3-NP + saline-administration markedly increased the activation of JNK, ERK, p38 MAPKs, and NF-*κ*B signaling pathways in striatum, compared to normal mice (Figure 7). However, the upregulation of the MAPKs and NF-*κ*B signaling pathways were significantly reduced by KRGE (250 mg/kg)-administration for 10 days (Figure 7). The results suggest that preadministration of KRGE may be involved in protective action for 3-NP-induced striatal toxicity by regulating the MAPKs and NF-*κ*B signaling pathway.

### 3.6. SB203580 and PD98059 Reduce 3-NP-Induced Lethality

**Figure 8:**
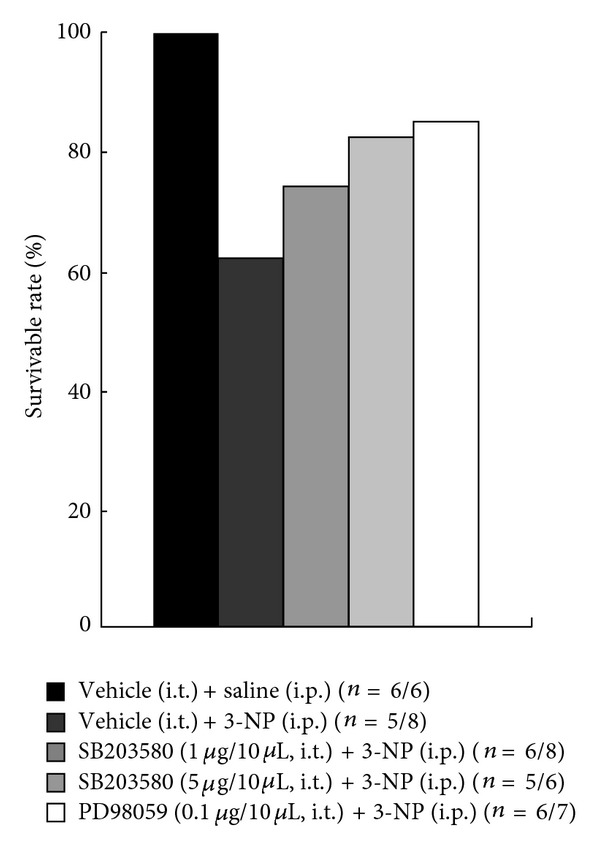
I.t. administration of SB203580 and PD98059 reduced 3-NP-induced lethality. Survival rate of 3-NP-administrated mice was increased by i.t. administration of SB203580 and PD98059.

After i.p. administration of 3-NP, elevated neurological impairment, lethality, striatal neuronal death, microglial activation, expression of inflammatory mediators, and activation of MAPKs and NF-*κ*B signal pathways were markedly reduced by the oral administration of KRGE (Figures 2–7). These results support the hypothesis that the inhibiting the activation of the MAPKs and NF-*κ*B signal pathways may reduce 3-NP-induced striatal toxicity. To address this issue, we directly introduced the representative p38 inhibitor (SB203580) and MEK inhibitor (PD98059) to the subarachnoid space of normal mice. Both inhibitors were introduced 20 minutes before the first and third 3-NP intoxication. In the vehicle-treated mice, the survival rate by 3-NP stimulation was 63% at 24 hours after last 3-NP injection. However, these survival rate were considerably increased by the i.t. preadministration of SB203580 (1.0 *μ*g, 75%; 5.0 *μ*g, 83%) or PD98059 (0.1 *μ*g, 86%) in a dose-dependent manner (Figure 8). These results indicate that i.t. introduction of SB203580 or PD98059 reduces 3-NP-induced lethality.

### 3.7. Individual Ginsenosides Reduce 3-NP-Induced Neurological Impairment and Lethality

**Figure 9:**
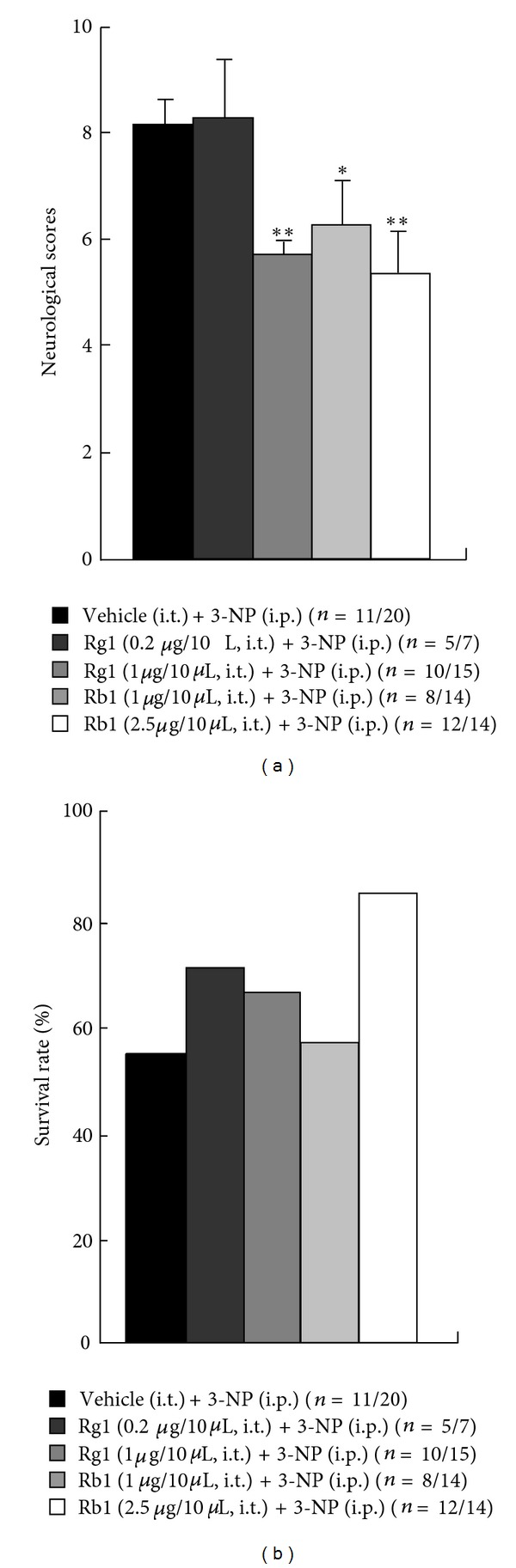
Individual ginsenosides reduce the 3-NP-induced neurological impairment (a) and lethality (b).

Additionally, to identify the active neuroprotective components in *P*. *ginseng*, we examined the effects of purified ginsenosides on 3-NP-induced neurological impairment and lethality. Individual ginsenoside-Rg1 and -Rb1 were selected for testing from ginsenosides that comprise the main components of ginseng. Rg1 and Rb1 have antioxidant or anti-inflammatory properties in nervous systems [33, 43, 53, 54]. We directly introduced the Rg1 and Rb1 to the subarachnoid space of normal mice as described above, and the i.p. injection of 3-NP was performed after 20 minutes. Interestingly, the i.t. preadministration of Rg1 (1.0 *μ*g) and Rb1 (1.0 and 2.5 *μ*g) reduced 3-NP-induced neurological impairment compared to vehicle-preadministrated mice. Preadministration of Rg1 (0.2 and 1.0 *μ*g) and Rb1 (2.5 *μ*g) increased 3-NP-induced survival rate compared to vehicle-preadministrated mice. (Figure 9). These results indicate that the i.t. introduction of Rg1 or Rb1 reduces 3-NP-induced neurological impairment and lethality.

### 3.8. Preadministration at High Dose of KRGE Does Not Induce Striatum Toxicity

Finally, to examine whether the administration of KRGE induces a toxic effect in the striatum, we treated normal mice with 250 and 500 mg/kg/day of KRGE for 12 days and assessed striatum toxicity. No evidence of a toxic effect was evident, compared to striatum of saline-administrated mice. Number of NeuN(+) cells, microglial and astroglial activation, number of TUNEL(+) cells, and expression of caspase-3 were not affected by the administration of KRGE (Supplementary Data 2).

## 4. Discussion

In the present study, we investigated whether KRGE can attenuate neurological impairment and striatal cell death in a neurodegeneration model induced by the administration of the mitochondrial toxin 3-NP. The preadministration of KRGE reduced lesion volumes and striatal degeneration, which were accompanied with the attenuation of neurologic deficits. Moreover, preadministration of KRGE inhibited microglial activation, expression of proinflammatory cytokines (TNF-*α*, IL-1*β*, and IL-6) and iNOS, and activation of the JNK, ERK, p38 MAPKs, and NF-*κ*B signaling pathways. The results suggest that the KRGE may attenuate 3-NP-induced striatal toxicity by inhibiting the activation of MAPKs and NF-*κ*B signaling pathways in the striatum.

Systemic 3-NP-administration inhibits mitochondrial succinate dehydrogenase- (SDH-) complex II and Ca^2+^ homeostasis [55–58], induces reproducible striatal degeneration, and causes motor deficits such as incoordination, brief hyperactivity followed by hypoactivity, dystonia and gait abnormalities [47], which are commonly observed in neurodegenerative disorders in including HD [59]. Thus, 3-NP has been widely used model HD [55, 56, 60]. In this study, the 3-NP + saline-administrated mice showed severe neurological deficits and striatal neuronal death, has been reported previously [55, 56, 60]. Preadministration of KRGE, on the other hand, markedly reduced the neurological deficits associated with a reduction in striatal neuronal death. Currently, no medical therapy has been proven to ameliorate these devastating clinical manifestations and, thus, our results indicate the feasibility of treatment in HD. Additionally, we compared the neuroprotective effect of KRGE among pre- (10 days before 3-NP injection), co- (at the same time with 3-NP injection), and postadministration (at the peak point of neurological impairment by 3-NP injection). Preadministration of KRGE showed the most neuroprotective effect in neurological impairment and striatal neuronal death, however, postadministration did not improve the neurological impairment. The results indicate that the intake of KRGE may delay or even prevent the development of HD-like syndrome.

It has recently been proposed that noncell autonomous neurodegeneration is an important event in the progression of different neurological disorders including HD [6, 7]. But, the beneficial or detrimental effect of reactive microglia and astrocyte remains contentious. Microglia comprise the major intrinsic immunocompetent phagocytic cells in the CNS. They are normally found in a resting form with spidery processes. Upon exposure of the CNS to any form of insult, such as infection, trauma, ischemia, or the presence of abnormal protein aggregations, resting microglia rapidly become activated and produce proinflammatory cytokines. In the case of HD, activated microglia release neurotrophic or neurotoxic mediators, which can be beneficial or toxic in neurodegenerative lesions [6, 7]. Therefore, controlling microglial activation may be an attractive therapeutic strategy for the treatment of various neurological disorders, including HD [6]. Administration with minocycline delayed mortality in a R6/2 mice model of HD and can be neuroprotective [36, 61] by inhibiting microglial activation, indicating a deleterious action of activated microglia on neuronal cells. Pyruvate blocks quinolinic acid-induced striatal toxicity by inhibiting microglial activation [3]. It was recently reported that KRGE and GTS attenuate the LPS-induced expression of iNOS, matrix metalloproteinase-9, and the proinflammatory cytokines TNF-*α*, IL-1*β*, and IL-6 in cultured microglial cells, and that ginsenoside Rh1-3 and compound K can reduce LPS-induced iNOS and cytokine expressions [41, 42]. Presently, preadministration of KRGE inhibited 3-NP-induced microglial activation (Iba-1-IR and the mRNA expression of OX-42), and the expression of proinflammatory cytokines (TNF-*α*, IL-1*β*, and IL-6) and iNOS. The results suggest that the administration of KRGE has an anti-inflammatory effect for 3-NP-induced striatal toxicity.

3-NP alters the activation of MAPKs signaling pathways in the striatum, and 3-NP-induced striatal degeneration is controlled by the activation of these pathways [11, 12]. JNK has been related to the induction of neuronal apoptosis in different experimental models [15–17]. Basal level of activated JNK was elevated in the striatum from presymptomatic yeast YAC128 transgenic mice [15]. In 3-NP experimental models, JNK is activated in striatal neuronal cells [16, 19] and in hippocampal cells [17, 18] via mechanisms similar to those observed in neuronal cell death induced by mutation *Htt* [18, 20]. It was recently reported that the LPS-induced phosphorylation of JNK in BV2 microglial cells can be considerably decreased by KRGE and GTS treatment [42]. Also, ginsenoside Rg1 can protect the substantia nigra (SN) neurons in 1-methyl-4-phenyl-1,2,3,6-tetrahydropyridine- (MPTP-) induced C57BL/6 mice by inhibiting the phosphorylation of JNK [53]. In this study, the 3-NP-induced activation of JNK was significantly decreased by preadministration of KRGE, consistent with previous reports.

ERK1/2 are crucial for the normal development and functional plasticity of the CNS [22]. Although the role of JNK in mutant *Htt*-associated toxicity seems relatively straightforward, the role of ERK activation in neurodegenerative diseases, including HD, is more complex, and may protect or damage neuronal cells [20–22]. Although the nature and extent of cellular injury may change the ultimate results of ERK activation, at least in HD, ERK activation protects against mutant *Htt*-associated toxicity [20]. It has recently been reported that KRGE and GTS markedly decrease the LPS-induced phosphorylation of ERK in BV2 microglial cells [42]. Also ginsenoside Rg1 can protect 6-OHDA-induced toxicity in MES23.5 cells by inhibiting the phosphorylation of ERK1/2 [54] and reduce the cytotoxicity in H_2_O_2_-induced PC12 cells expressing mutant *Httex1* under the control of an inducible promoter by blocking the ERK1/2 activation [16]. In the present study, the increased phosphorylation of ERK was downregulated by the preadministration of KRGE for 10 days, and the i.t. administration of MEK inhibitor PD98059 increased the survival rate of mice with 3-NP injection. The results suggest that control of phosphorylation of ERK may be a potential approach to HD therapy.

Striatal p38 is activated by reactive oxygen species [62–64] and its crosstalk with caspases [65]. Both reactive oxygen species and caspase activity can be increased as a result of altered calcium homeostasis induced by mutant *Htt* early in HD [66, 67]. Basal levels of activated p38 are upregulated in the striatum from presymptomatic yeast YAC128 transgenic mice [15]. In a quinolinic acid model of excitotoxicity, p38 was activated in the core region within 1 hour of injection in a pattern consistent with p38 activation preceding cell death [23]. Furthermore, p38 is activated in striatal neurons of R6/2 mice at a late stage in HD [24]. Interestingly, KRGE and GTS treatment can markedly decrease the LPS-induced phosphorylation of p38 in BV2 microglial cells [42]. In the present study, the preadministration of KRGE blocked the phosphorylation of p38 in striatum stimulated with 3-NP. Additionally, i.t. preadministration of p38 inhibitor SB203580 increased the survival rate of mice with 3-NP-induced striatal toxicity. The results indicate that regulating the phosphorylation of p38 in the striatum may have positive potential for HD-like syndromes.

The NF-*κ*B pathway also plays a crucial protective role in neurodegeneration. Previous studies demonstrated that NF-*κ*B pathways modulate iNOS, several cytokines, and matrix metalloproteinase-9 expression in LPS-stimulated microglia [68–70], and that KRGE and GTS suppress NF-*κ*B pathways [42]. The present finding that KRGE significantly suppress NF-*κ*B activity in 3-NP-induced striatum indicate that the inhibition of NF-*κ*B signaling pathways might be correlated with the anti-inflammatory effects of KRGE.

## 5. Conclusions

While increased striatal MAPKs and NF-*κ*B phosphorylation are important for HD-like syndrome based on the p38 inhibitor and MEK inhibitor studies, the direct link between KRGE's inhibitory effects in 3-NP-induced neurological impairment and its modulating effects on MAPKs and NF-*κ*B activation has not been established. In this study, KRGE attenuated neurological impairment, striatal neuronal death, microglial activation, several cytokines, iNOS, and activation of MAPKs and NF-*κ*B pathways in a 3-NP-induced HD model. The i.t. introduction of SB203580 and PD98059 reduced the lethality by 3-NP injection. These results strongly suggest that KRGE has neuroprotective and an anti-inflammatory effects on 3-NP-induced striatal toxicity by inhibiting the striatal MAPKs and NF-*κ*B activation in the striatum.

## Supplementary Material

Immunohistochemical Evaluation: Immunohistochemistry and detection of NeuN, Iba-1, GFAP, capase-1 were performed as described in Materials and Methods of manuscript. *In situ* detection of fragmented DNA (terminal deoxynucleotidyl transferase-mediated UTP nick end labeling, TUNEL). The fragmentation of DNA was examined using an ApopTag® Peroxidase In situ Apoptosis Detection Kit (S7100) (Millipore, U.S.A.) according to the manufacturer's instructions. Briefly, brain sections were placed to enzymatic digestion with a 20 *μ*g/ml of proteinase K for 5 minutes, treated with 5% H_2_O_2_ for 20 minutes to exhaust endogenous peroxidase activity, and washed with PBS (0.1 M, pH 7.4). They were then immersed in an ApopTag® Equilibration Buffer to label the 3'-OH ends of fragmented DNA for 10 minutes and incubated with terminal deoxynucleotidyl transferase enzyme at 37°C for 1 hour. After washing with PBS, sections were incubated with anti-digoxygenin conjugated peroxidase and the peroxidase substrate (DAB) to detect signs of apoptotic cell death.
Western blot analysis: Western blot analysis of capase-3 was performed as described in Materials and Methods of manuscript.

## Figures and Tables

**Table 1:** Analytical data received from the Korean Ginseng Corporation on the contents of KRGE.

Ingredients	Content (mg/g)
Organic (58%)	
Clude protein (11.1%)	111.3
Clude saponin (7.5%)	75.0
Carbohydrate (23.9%)	
Ginseng acid polysaccharide	6.5
Clude fat	0.6
Free saccharide	110.2
Clude fiber	121.6
Nitrogen compound (1.8%)	
Amino acid	9.5
Arginine-fructose-glucose	8.7
Phenolic compounds (0.09%)	0.9
Other (13.5%)	135.8
Inorganic (42%)	
Mineral (5.5%)	55.0
Water (36.5%)	365.0

Total (100%)	1000.0

**Table 2:** PCR primer sequence for PCR analysis.

	Forward primer (5′→3′)	Reverse primer (5′→3′)
TNF-*α*	AGC AAA CCA CCA AGT GGA GGA	GCT GGC ACC ACT AGT TGG TTG T
IL-1*β*	TTG TGG CTG TGG AGA AGC TGT	AAC GTC ACA CAC CAG CAG GTT
IL-6	TCC ATC CAG TTG CCT TCT TGG	CCA CGA TTT CCC AGA GAA CAT G
iNOS	GGC AAA CCC AAG GTC TAC GTT	TCG CTC AAG TTC AGC TTG GT
HO-1	CCT GGA GCA GGA CAT GGC	AAT CAT CCC TTG CAC GCC
OX-42	GAC CGG TGC AGG ATC ATA CAG	CCT TTC GTC CGC TTC AGA GT
GAPDH	AGG TCA TCC CAG AGC TGA ACG	CAC CCT GTT GCT GTA GCC GTA T
