# Supplementary material for: Korean Red Ginseng Extract Attenuates 3-Nitropropionic Acid-Induced Huntington's-Like Symptoms
Source: Evid Based Complement Alternat Med. 2013 Jan 27;2013:237207. doi: 10.1155/2013/237207 (PMC3568869; doi:10.1155/2013/237207)

Supplementary Data 1

Treatment	Group	Neurodeficit score											Total number	Survival rate (%)	Lesioned mice; No. (%)
		0	1	2	3	4	5	6	7	8	9	10			
Pre-administration (- 10 day)	Normal	10											10	10 (100.0)	0 (0.0)
	3-NP+saline							3	6	0	3	3	31	15 (48.4)	6 (40.0)
	3-NP+KRGE 50						2	2	5	2	2	1	18	14 (77.8)	3 (21.4)
	3-NP+KRGE 100				2	5	4	2					16	13 (81.3)	2 (15.3)
	3-NP+KRGE 250		2	2	4	5							15	13 (86.7)	1 (7.7)
	Saline+KRGE 250	3											3	3 (100.0)	0 (0.0)
Co-administration (0 day)	Normal	10											10	10 (100.0)	0 (0.0)
	3-NP+saline							5	3	1	2	2	31	13 (41.9)	8 (61.5)
	3-NP+KRGE 50						2	4	3	1	0	2	18	12 (66.7)	4 (33.3)
	3-NP+KRGE 100					1	1	6	2	1			16	11 (68.8)	3 (27.3)
	3-NP+KRGE 250				1	3	1	2	4				15	11 (73.3)	2 (18.2)
	Saline+KRGE 250	3											3	3 (100.0)	0 (0.0)
Peak time administration (2 day)	Normal	10											10	10 (100.0)	-
	3-NP+saline					3	1						8	4 (50.0)	-
	3-NP+KRGE 50				1	2	1						8	4 (50.0)	-
	3-NP+KRGE 100				1	4							8	5 (62.5)	-
	3-NP+KRGE 250				1	3	1						8	5 (62.5)	-
	Saline+KRGE 250	3											3	3/3(100)	-

Supplementary Data 2

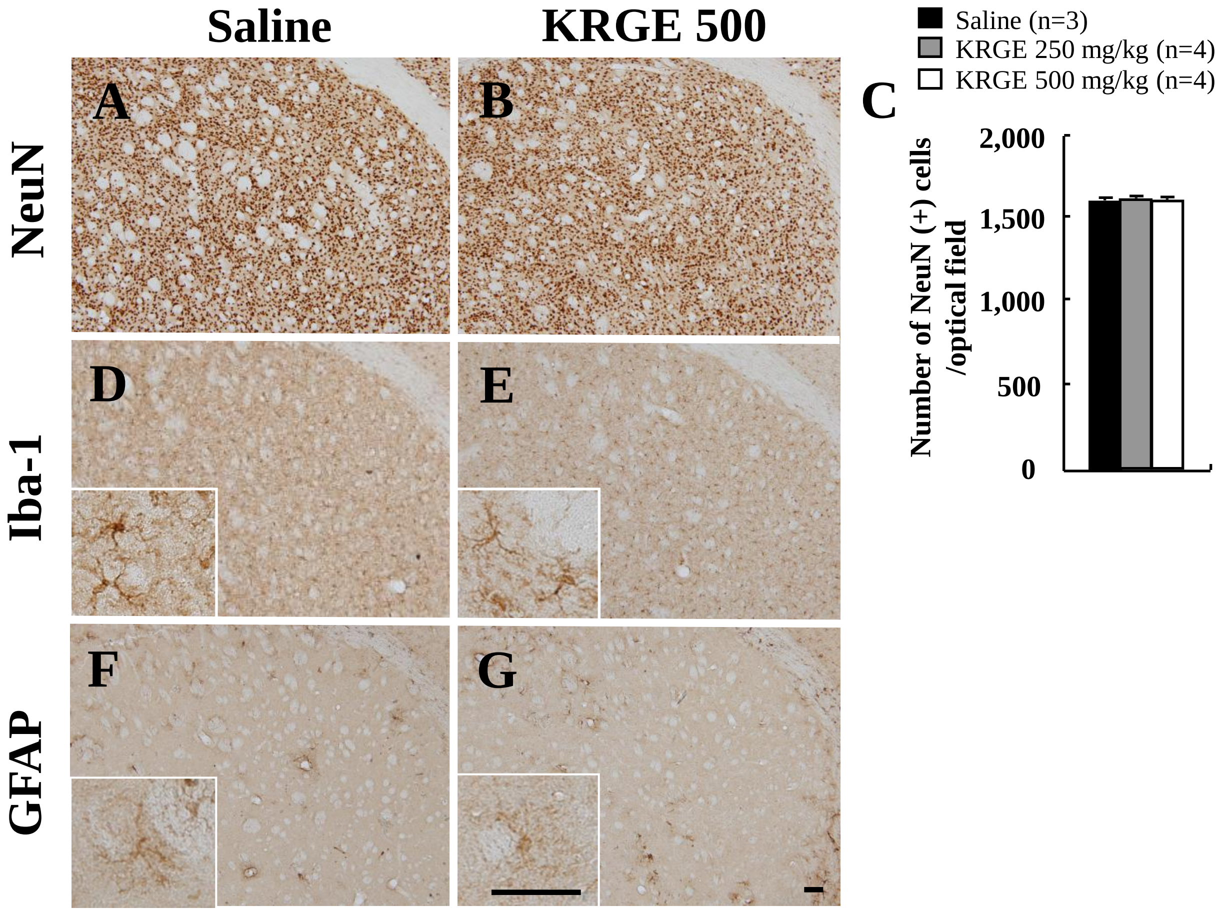

Supplementary Data 2 – continued

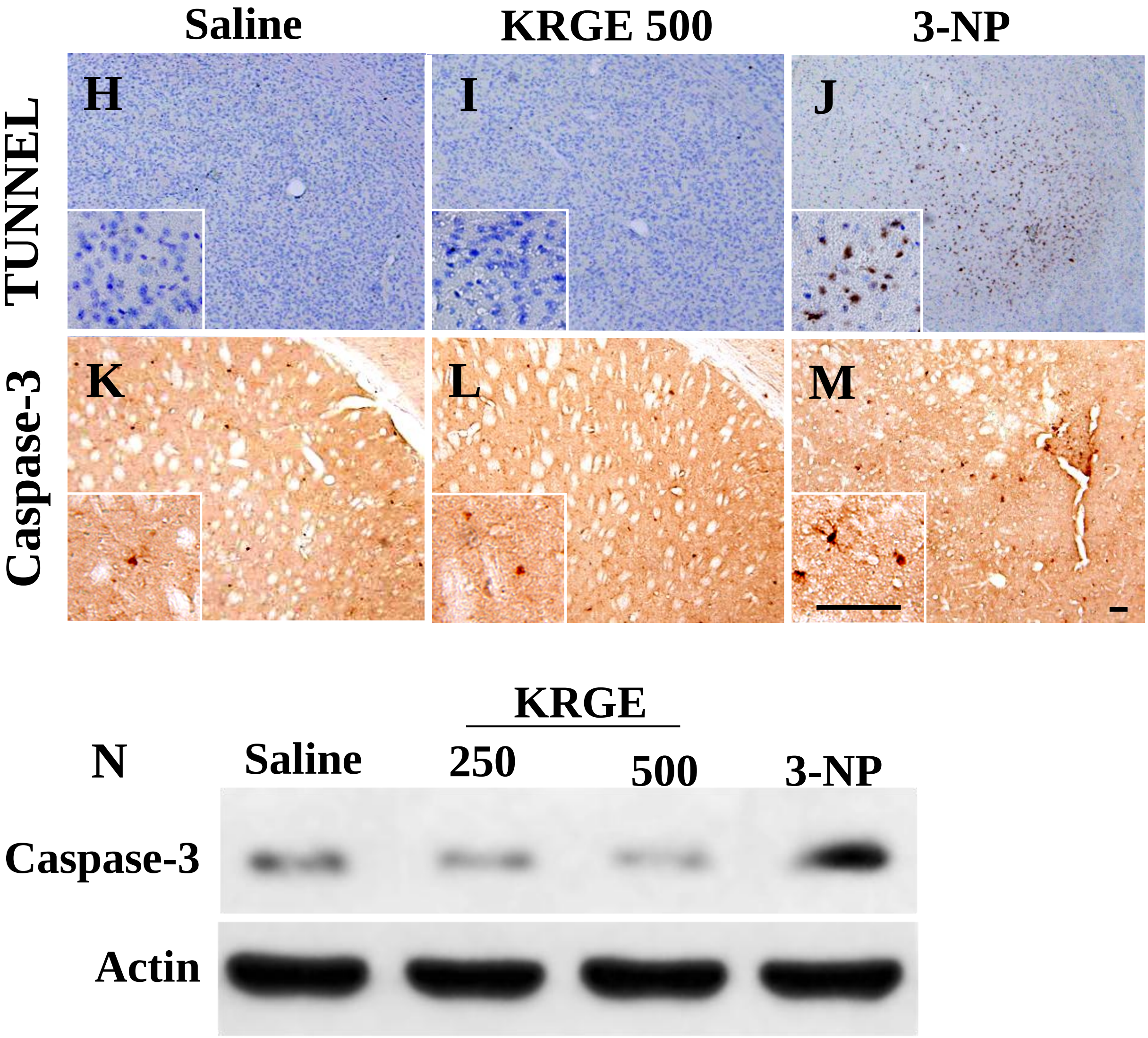

Supplement: Supplementary file 1 — Immunohistochemical Evaluation: Immunohistochemistry and detection of NeuN, Iba-1, GFAP, capase-1 were performed as described in Materials and Methods of manuscript. In situ detection of fragmented DNA (terminal deoxynucleotidyl transferase-mediated UTP nick end labeling, TUNEL). The fragmentation of DNA was examined using an ApopTag® Peroxidase In situ Apoptosis Detection Kit (S7100) (Millipore, U.S.A.) according to the manufacturer's instructions. Briefly, brain sections were placed to enzymatic digestion with a 20 μg/ml of proteinase K for 5 minutes, treated with 5% H2O2 for 20 minutes to exhaust endogenous peroxidase activity, and washed with PBS (0.1 M, pH 7.4). They were then immersed in an ApopTag® Equilibration Buffer to label the 3'-OH ends of fragmented DNA for 10 minutes and incubated with terminal deoxynucleotidyl transferase enzyme at 37°C for 1 hour. After washing with PBS, sections were incubated with anti-digoxygenin conjugated peroxidase and the peroxidase substrate (DAB) to detect signs of apoptotic cell death. Western blot analysis: Western blot analysis of capase-3 was performed as described in Materials and Methods of manuscript. [file 237207.f1.pdf]
